# Re-interpreting mesenteric vascular anatomy on 3D virtual and/or physical models, part II: anatomy of relevance to surgeons operating splenic flexure cancer

**DOI:** 10.1007/s00464-022-09394-5

**Published:** 2022-06-30

**Authors:** Bjarte Tidemann Andersen, Bojan V. Stimec, Airazat M. Kazaryan, Peter Rancinger, Bjørn Edwin, Dejan Ignjatovic

**Affiliations:** 1 Department of Gastrointestinal Surgery, Østfold Hospital Trust, Grålum, Norway; 2 Institute for Clinical Medicine, University of Oslo, Oslo, Norway; 3 Anatomy Sector, Teaching Unit, Faculty of Medicine, University of Geneva, Geneva, Switzerland; 4 Interventional Centre, Oslo University Hospital - Rikshospitalet, Oslo, Norway; 5 Department of Surgery, Fonna Hospital Trust, Odda, Norway; 6 Department of Faculty Surgery, I.M. Sechenov First, Moscow State Medical University, Moscow, Russia; 7 Department of Surgery N 2, Yerevan State Medical University after M. Heratsi, Yerevan, Armenia; 8 Department of Gastrointestinal and Pediatric Surgery, Oslo University Hospital-Rikshospitalet, Oslo, Norway; 9 Department of Digestive Surgery, Akershus University Hospital, Lørenskog, Norway

**Keywords:** Splenic flexure cancer, Left colectomy, D3 mesenterectomy, Middle colic artery, Inferior mesenteric artery, Mesenteric vascular anatomy

## Abstract

**Background:**

The splenic flexure is irrigated from two vascular areas, both from the middle colic and the left colic artery. The challenge for the surgeon is to connect these two vascular areas in an oncological safe procedure.

**Materials and methods:**

The vascular anatomy, manually 3D reconstructed from 32 preoperative high-resolution CT datasets using Osirix MD, Mimics Medical and 3-matic Medical Datasets, were exported as STL-files, video clips, stills and supplemented with 3D printed models.

**Results:**

Our first major finding was the difference in level between the middle colic and the inferior mesenteric artery origins. We have named this relationship a mesenteric inter-arterial stair. The middle colic artery origin could be found cranial (median 3.38 cm) or caudal (median 0.58 cm) to the inferior mesenteric artery. The lateral distance between the two origins was 2.63 cm (median), and the straight distance 4.23 cm (median). The second finding was the different trajectories and confluence pattern of the inferior mesenteric vein. This vein ended in the superior mesenteric/jejunal vein (21 patients) or in the splenic vein (11 patients). The inferior mesenteric vein confluence could be infrapancreatic (17 patients), infrapancreatic with retropancreatic arch (7 patients) or retropancreatic (8 patients). Lastly, the accessory middle colic artery was present in ten patients presenting another pathway for lymphatic dissemination.

**Conclusion:**

The IMV trajectory when accessible, is the solution to the mesenteric inter-arterial stair. The surgeon could safely follow the IMV to its confluence. When the IMV trajectory is not accessible, the surgeon could follow the caudal border of the pancreas.

**Graphical abstract:**

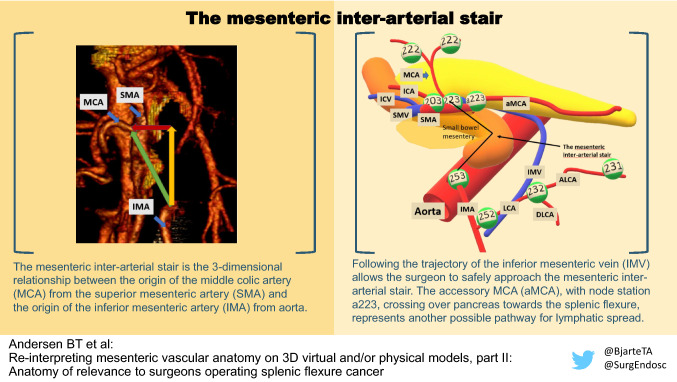

Splenic flexure cancer has historically presented a challenge for surgeons. Numerous techniques were described, long-term outcomes for patients have been reported as poorer and complications higher when compared to other colon segments [1–3]. Comparable survival rates have been recently published [4, 5].

The work of Coffey et al. describes the mesentery as a continuous organ [6]. The vessels of interest to the surgeon operating splenic flexure cancer are the middle colic artery (MCA), arising from the superior mesenteric artery (SMA) and the left colic artery (LCA), arising from the inferior mesenteric artery (IMA). Up to date a D3 extended mesenterectomy for splenic flexure cancer is missing a clear definition for the border between these two irrigation areas, i.e., between the left colonic mesentery and that of the right colon and small bowel [7]. In surgical terms this area of the mesentery between the above-mentioned vessels connects the origins of these arteries and holds the solution for a personalized lymphadenectomy. Moreover, these two vascular beds lie in different planes of the human body, the small bowel mesentery positioned between them, in this manner further complicating its identification [8].

The aim of this study is to determine the three-dimensional anatomical relationship between the origins of the two separate vascular beds irrigating the splenic flexure, namely the vessels of interest to the surgeon operating D3 extended mesenterectomy for the splenic flexure cancer. The further aim is to identify and clearly define the surgical challenges (level and distance between vessel origins; vascular dominance) when accessing the mesenteric junction of these two vascular beds.

## Materials and methods

### CT datasets

Preoperative computed tomography (CT) datasets from 32 patients were included. The datasets were derived from the prospective multicentre “Safe Radical D3 Right Hemicolectomy for Cancer through Preoperative Biphasic MDCT Angiography” trial (Norwegian Ethical Committee approval REK 2010/3354 and ClinicalTrial Identifier NCT01351714). Different results from the same dataset have been presented in our previous article Re-interpreting mesenteric vascular anatomy on 3D virtual and/or physical models [9].

The CT datasets were high resolution (1 mm slab and less) and included a morphologically intact left colon with its vascular tree. The portovenous phase datasets were subjected to a detailed manual segmentation and morphometry using three image processing software: FDA approved Osirix MD v. 12.0.1 (Pixmeo, Bernex, Switzerland), Mimics Medical image processing software, ver. 22.0, and 3-matic Medical software, ver. 14.0, both Windows 7 ultimate edition × 64 2017 (Materialise NV, Leuven, Belgium).

### Image processing

We have previously published our protocol for manual editing and 3D reconstruction [10]. The left branch of the MCA, the accessory MCA (aMCA) if present, the middle colic vein (MCV), the suprapelvic IMA and the inferior mesenteric vein (IMV) were defined as Regions Of Interest (ROI)s. The vascular arborisation was enhanced by validating pixels outside ROIs to air and revalidating pixels inside ROIs to their original value, thus erasing the surrounding tissues.

All datasets were subjected to manual thresholding (with profile line) for attributing value to voxels of all vascular structures. Using the Osirix scout files, the initial mask was first cropped, then single and multiple slices editing with interpolation was done. After this process, a 3D object mask was calculated for the vessels.

These 3D object masks were exported as STL (stereolithography) format for use in 3D printing, and as MXP format for use in virtual measuring in 3-matic. The MXP files were finally exported as 3D PDF with annotations on vascular structures.

### Morphological and morphometric analyses

All distances were measured on 3D virtual models in Osirix using a length tool.

**Fig. 1:**
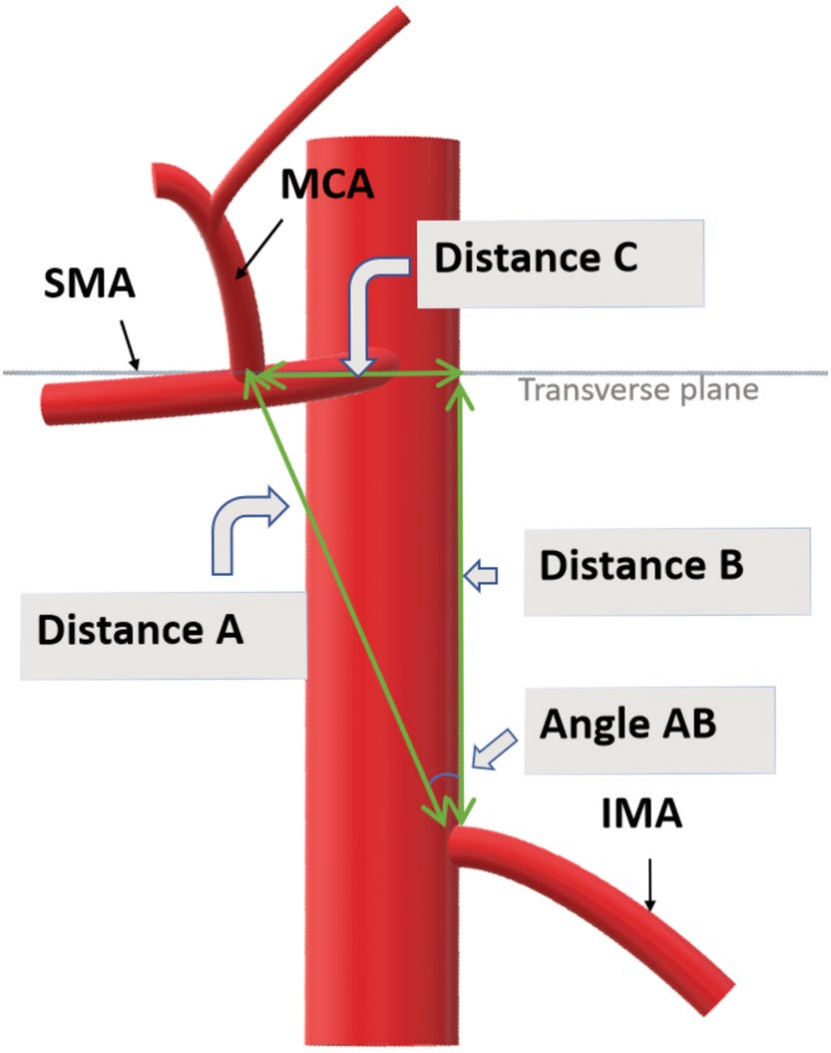
Distance A: From the MCA origin to the IMA origin. Distance B: Parallel to the aorta axis from the IMA origin to the transverse plane through the MCA origin. Distance C: In the transverse plane from the MCA origin to the intersection with Distance B. The angle between the Distance A and Distance B intersection was named Angle AB Vessels *MCA* middle colic artery, *SMA* superior mesenteric artery, *IMA* inferior mesenteric artery

The difference in level between the MCA origin and the IMA origin was named a mesenteric inter-arterial stair. The values measured and the relationship between the values are clarified in Fig. 1.

Distance A (oblique distance) was measured from the MCA origin to the IMA origin. Distance B (longitudinal distance) was measured parallel to the aorta axis from the IMA origin to the intersection with the transverse plane through the MCA origin. Distance C (lateral distance) and the angle AB were calculated using the Pythagorean theorem. These lines form a right-angled triangle with the MCA origin in one and the IMA origin in the other vertex.

The angle AB represents the direction, distance A (oblique distance) represents the length, and distance C (lateral distance) represents the height of the mesenteric inter-arterial stair.

The distance from the IMA origin to the LCA origin was measured on the IMA in a stepwise manner along the vessel. Calibres of the vessels were measured at their base, using a length tool, and measuring the largest diameter.

### The vessels

#### SMA

The second ventral visceral branch from the abdominal aorta.

#### MCA

Arises from SMA, enters and supplies the transverse mesocolon.

#### Accessory MCA

A vessel arising from SMA cranial to the proper MCA, entering the transverse mesocolon and running towards the splenic flexure.

#### LCA

The first left branch of the IMA, supplying the descending colon, the splenic flexure and the transverse colon.

#### IMV

Drains the left colon. The position of the IMV confluence was categorized according to the vessel it drained into, i.e., superior mesenteric vein (SMV)/ jejunal vein (JV) or the splenic vein (SV). The surgical accessibility of the IMV confluence was assessed according to its trajectory in relation to the pancreas as: infrapancreatic (accessible), infrapancreatic with a retropancreatic arch (partially accessible) or retropancreatic (not accessible).

### Vascular dominance of the splenic flexure

Estimations about the vascular dominance at the splenic flexure were made after examination of the CT datasets and the 3D CT virtual models for branching patterns, the intensity of contrast in the vessels and the calibre of the feeding and draining blood vessels. The vascular dominance was categorized in three groups; namely, predominately SMA through MCA/aMCA and drained through MCV, predominately IMA through ALCA and drained through IMV, or co-dominance from both vascular areas.

### 3D models

The physical 3D models were printed using the Ultimaker S3 (Ultimaker B.V., Utrecht, The Netherlands) fused filament fabrication printer. The stl-file was opened with Ultimaker Cura 4.9.1 software. The printer setting was set to profile fine (0.1 mm). After slicing the 3D model was printed with Ultimaker Pearl-White PLA (polyactic acid) with supports made by water-soluble Ultimaker PVA natural (polyvinyl alcohol). All models were printed in 1:1 scale. Depending on the complexity of the model, the printing process took from 24 to 76 h. After cooling, the model is removed and left in a container with water for several hours.

**Fig. 2:**
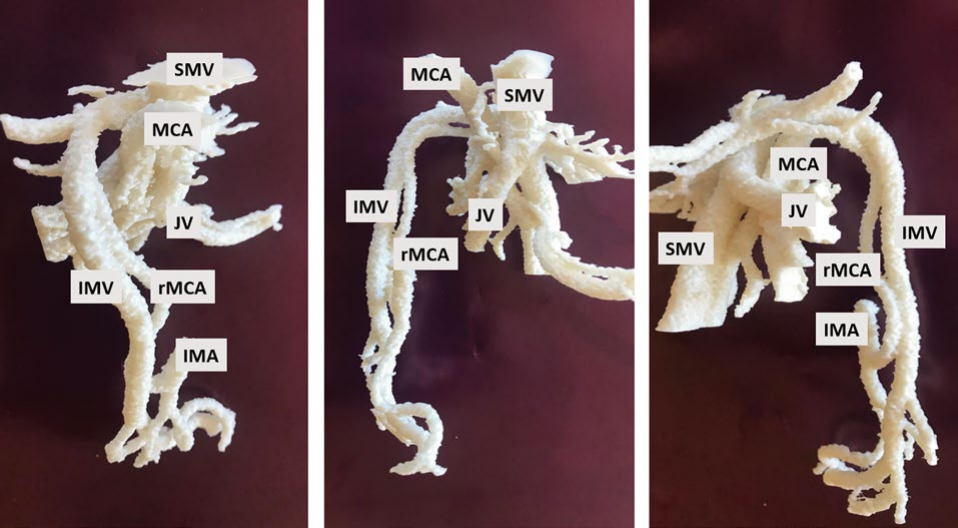
The 3D-printed vascular model gives valuable visual and tactile information as the surgeons can view the anatomy from different angles while holding the model in her/his own hands

Because of the cost and time-consuming process, the 3D printed models were used for selected difficult cases to further visualize the IMA and LCA branching pattern as shown in Fig. 2.

### Statistics

Data from the pre-operative abdominal CT datasets were analysed using the IBM SPSS Statistics for Windows Version 27.0. (2020), Armonk, NY: IBM Corp. For continuous variables, the data are presented as median (range)/mean ± standard deviation. For categorical variables, the data are presented as numbers (percent). The Shapiro–Wilk test was used to test normality in the distributions. The Mann–Whitney test was applied to examine differences between morphometric subgroups for non-normal distributed values. The Independent Samples *t*-Test was used for subgroups with normal distributed values.

## Results

### Virtual and physical models

Thirty-two 3D virtual and 15 physical 3D printed vascular models were created from the CT datasets, acquired from 19 female and 13 male patients (total 32 patients) mean age 67.4 ± 7.5 years.

### The inferior mesenteric artery and the mesenteric inter-arterial stair

IMA was present in all patients (calibre 0.43 (0.20–0.85)/0.44 ± 0.12 cm).

Twenty-eight of the patients had the MCA origin cranial to the IMA origin, implying a positive value for Distance B (longitudinal). Three of the patients had the MCA origin caudal to the IMA origin, implying a negative value for Distance B (longitudinal).

**Fig. 3:**
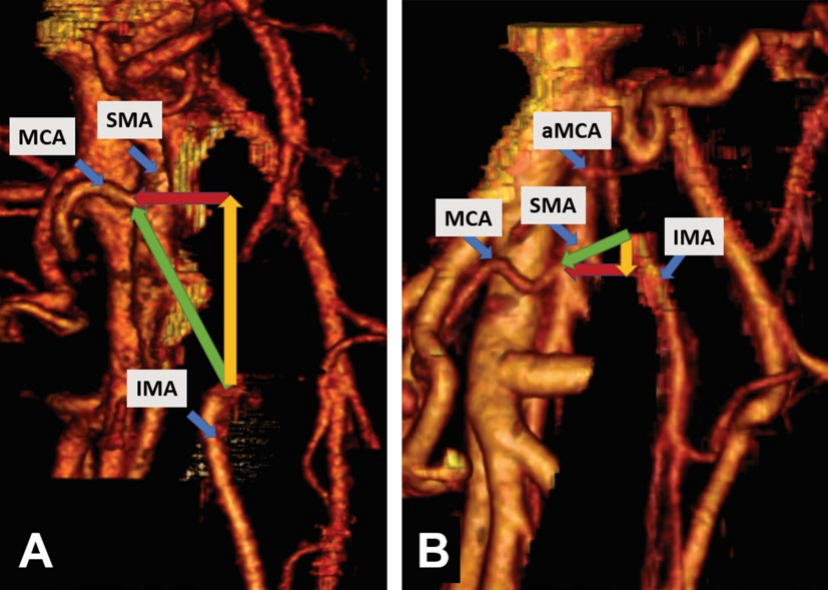
A CT reconstruction visualizing the mesenteric inter-arterial stair in two patients. **A** An MCA origin cranial to the IMA origin implies a positive Distance B (yellow) and that Distance A (green) points from the IMA origin cranioventrally towards the MCA origin. Distance C (red) represents the height of the stair. In this patient Distance A was 4.23 cm, Distance B 4.07 cm, Distance C 1.16 cm and the angle between Distance A and B 15.9 degrees. **B** An MCA origin caudal to the IMA origin implies a negative Distance B (yellow) and that Distance A (green) points caudoventrally. In this patient Distance A was 2.34 cm, Distance B − 0.58 cm, Distance C 2.26 cm and the angle between Distance A and B 75.6 degrees Vessels *aMCA* accessory middle colic artery, *MCA* middle colic artery, *SMA* superior mesenteric artery, *IMA* inferior mesenteric artery (Color figure online)

The mesenteric inter-arterial stair is presented in Fig. 3. The distances and angles are presented for all patients first and then separately for patients with the MCA origin cranial and caudal to the IMA origin in Table 1.

**Table 1:** The distances and angles for all patients and subgroups of patients with the MCA origin cranial and caudal to the IMA origin

	All patients	The MCA origin cranial to the IMA origin (*n* = 28)	The MCA origin caudal to the IMA origin (*n* = 3)
Distance A (oblique)	4.23 (2.09–10.20) /4.31 ± 1.80 cm	4.30 (2.13–10.20) /4.53 ± 1.75 cm	2.30 (2.09 to 2.34) /2.24 ± 0.14 cm
Distance B (longitudinal)	2.87 (− 1.04–9.65) /3.05 ± 2.30 cm	3.38 (0.36–9.65) /3.46 ± 2.03 cm	− 0.58 (− 1.04 to − 0.55) /− 0.72 ± 0.28 cm
Distance C (lateral)	2.63 (0.85–4.74) /2.56 ± 0.88 cm	2.71 (0.85–4.74) /2.61 ± 0.91 cm	2.05 (2.01 to 2.26) /2.11 ± 0.13 cm
Angle AB	39.9° (11.8°–82.8°) /43.6 ± 20.1°	35.4° (11.8°–82.8°) /40.6° ± 18.8°	74.9° (63.1° to 75.8°) /71.2° ± 7.1°

Values are given as median (range) / mean ± standard deviation

*MCA* middle colic artery, *IMA* inferior mesenteric artery

The patients with the MCA origin cranial to the IMA origin had sharper angle AB (Student *t*-Test *P* = 0.010) between Distance A (oblique) and Distance B (longitudinal) compared to the patients with the MCA origin caudal to the IMA origin. Furthermore, Distance A (oblique) was longer (Mann–Whitney *P* = 0.002), as well as Distance B (longitudinal) (Mann–Whitney *P* = 0.006), while Distance C (lateral) was only slightly longer (Student *t*-Test *P* = 0.361) when the MCA origin was cranial to the IMA origin.

All patients with the MCA origin caudal to the IMA origin had an accessory MCA.

### MCA and accessory MCA

MCA was present in all patients (calibre: 0.28 (0.18–0.40)/0.29 ± 0.06 cm), branching off IMA in one and SMA in 31 patients. The MCA bifurcation was most often located in front of SMV, i.e., in 17 patients. The aMCA was found in ten patients, following the IMV near the caudal pancreatic border in five of the patients [9].

### Vascular dominance of the splenic flexure

The splenic flexure was predominantly supplied from IMA/ALCA in 19 patients. Eight patients had predominantly supply from SMV/MCA, and five were supplied equally form both sides.

### The inferior mesenteric vein

**Fig. 4:**
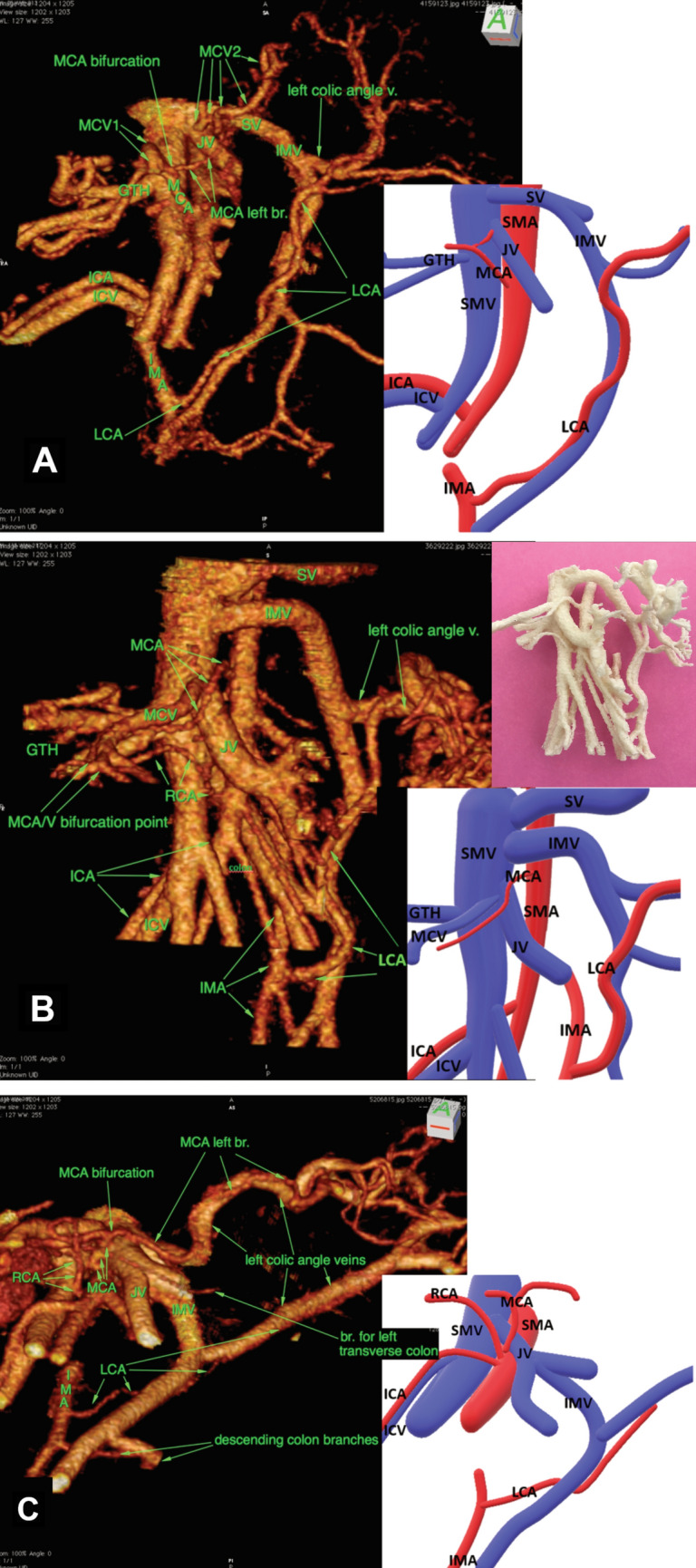
Confluence patterns of the inferior mesenteric vein (IMV) to the portal vein flow. Each pathway is illustrated with an actual reconstruction and a schematic illustration. In addition, a 3D printed model is shown for comparison in (Fig. 3**B**). **A** IMV joining the splenic vein (11 patients), **B** IMV joining the superior mesenteric vein (18 patients), **C** IMV joining a jejunal vein (three patients) Vessels *SMV* superior mesenteric vein, *SMA* superior mesenteric artery, *GTH* gastrocolic trunk of Henle, *MCV* middle colic vein, *MCA* middle colic artery, *RCA* right colic artery, *ICV* ileocolic vein, *ICA* ileocolic artery, *JV* jejunal vein, *SV* splenic vein, *IMV* inferior mesenteric vein, *IMA* inferior mesenteric artery, *LCA* left colic artery

**Fig. 5:**
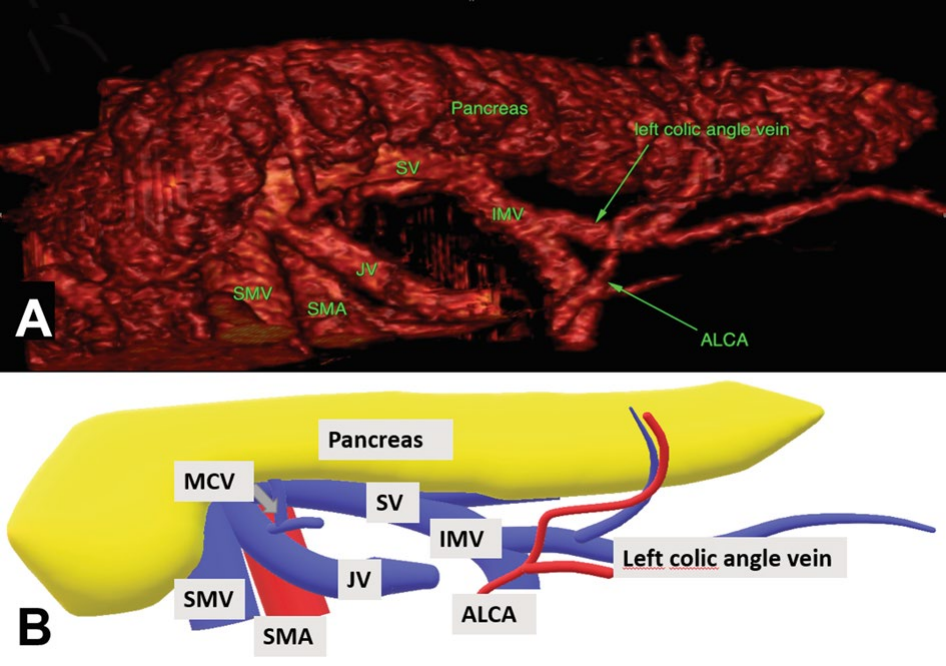
3D reconstruction (**A)** and schematic 3D illustration (**B)** showing the inferior mesenteric vein (IMV) confluence into splenic vein (SV). Note the trajectory of the splenic vein along the pancreatic border Vessels *SMV* superior mesenteric vein, *MCA* middle colic artery, *SMA* superior mesenteric artery, *JV* jejunal vein, *ALCA* ascending left colic artery

The IMV had its confluence to SV in 11 patients as shown in Fig. 4A, and into the drainage area of SMV in 21 patients. Of the latter group, 18 had an IMV draining directly into SMV as shown in Fig. 4B, while the remaining three drained to a jejunal vein as shown in Fig. 4C. No confluence into the SV and SMV junction were seen. The IMV confluence and its surgical accessibility were assessed as: infrapancreatic (accessible) 17 patients, infrapancreatic with a retropancreatic arch (partially accessible) seven patients or retropancreatic (not accessible) eight patients. When analysing the subgroup IMV confluences to the SV (*n* = 11), the majority (*n* = 7) were infrapancreatic (accessible) as shown in Fig. 5, while the minority (*n* = 4) were positioned retropancreatic (inaccessible). A breakdown is shown in Table 2.

**Table 2:** The IMV confluence and surgical accessibility

Confluence	Surgical accessibility			
	Infrapancreatic	Infrapancreatic with retropancreatic arch	Retropancreatic	Total
SV	7 (63.6)	0	4 (36.4)	11
SMV	7 (38.9)	7 (38.9)	4 (22.2)	18
JV	3 (100)	0	0	3
Total	17 (53.1)	7 (21.9)	8 (25.0)	32

Data are given as a number (percentage)

*SV* splenic vein, *SMV* superior mesenteric vein, *JV* jejunal vein

## Discussion

We have identified three major anatomical challenges surgeons face when attempting to perform D3 extended mesenterectomy for splenic flexure cancer.

**Fig. 6:**
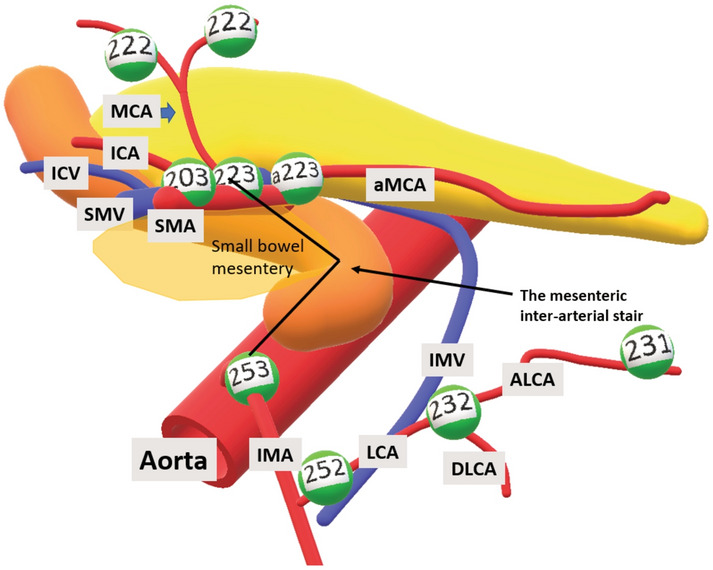
Illustration of “the mesenteric inter-arterial stair”. The stair (purple) ascends from the IMA to the MCA origin. The small bowel mesentery is marked with translucent blue, and the lymph node stations are numbered according to the Japanese classification [11], the central lymph node station belonging to the aMCA is additionally named a223 to distinguish it from 223 belonging to MCA Vessels *MCA* middle colic artery, *aMCA* accessory middle colic artery, *ICA* ileocolic artery, *ICV* ileocolic vein, *SMV* superior mesenteric vein, *SMA* superior mesenteric artery, *IMA* inferior mesenteric artery, *LCA* left colic artery, *IMV* inferior mesenteric vein, *ALCA* ascending left colic artery, *DLCA* descending left colic artery (Color figure online)

The first is “The mesenteric inter-arterial stair”, which signifies the difference in level and position between arterial origins of interest (MCA/IMA). The stair is illustrated in Fig. 6 with lymph node stations numbered according to the Japanese classification [11]. This “stair” represents a two- fold challenge; the first is to overcome the difference in the level of the “stair”, and the second is to avoid injury of the small bowel mesentery that contains the arterial and venous tributaries irrigating the small bowel.

In general, it is accepted that the mesentery is a continuous structure [6], but there is no consensus in the literature where these two above-mentioned vascular areas meet [5]. Perhaps even more important is to be aware that the small bowel mesentery lies positioned between the transverse and descending colon mesentery (directly between the above-mentioned arterial origins) as a consequence of the mesenteric rotation in the developing embryo [8, 12]. Our results imply that the solution for this border can be resolved through the trajectory of IMV, but here we will also find the second challenge, namely the different trajectories of IMV. This article shows that in 18 out of 32 patients IMV terminated directly into SMV, when including the IMVs with a confluence to a JV, the number is 21 out of 32. The remaining 11 out of 32 patients had their confluence into SV. Other studies have published data suggesting that the most common termination of SMV is into SV [13–15]. Compared to our findings, data from the literature show a slightly lower occurrence of the IMV termination into SMV, between 18.5% [14] and 40% [16]. In order to arrive to its confluence to SMV the IMV trajectory needs to climb the mesenteric inter-arterial stair and avoid the small bowel mesentery, in this way tracing the path of surgical dissection required for a safe and complete mesocolic excision. Consequently, it seems that surgical access to IMV is of the essence when attempting D3 extended mesenterectomy for splenic flexure cancer. The IMV confluence was accessible in most cases (infrapancreatic/infrapancreratic with loop) in our series and not readily accessible (retropancreatic) to the surgeon in only eight out of 32 patients. It should be noted that when the IMV confluence is to the splenic vein it is still accessible in the majority of cases since the splenic vein has a low trajectory along the inferior pancreatic border, as shown in Fig. 5. When this confluence is not accessible it seems that a corresponding line along the caudal border of the pancreas would provide a similar result, leading to SMV, and the MCA origin. Interestingly, we have not found in literature, including the latest systematic review [15], mentioning of surgical accessibility of the IMV confluence to SMV or SV.

The area of the mesenteric inter-arterial stair, between the MCA and the IMA origin, is of importance since the lymphatic drainage from the splenic flexure is not one-sided, as shown in the literature that lymph node metastases can be found along both vascular tributaries [17]. Our series shows that in 19 patients the splenic flexures were mainly supplied from IMA, in eight patients from MCA and in five patients equally from both vessels.

**Fig. 7:**
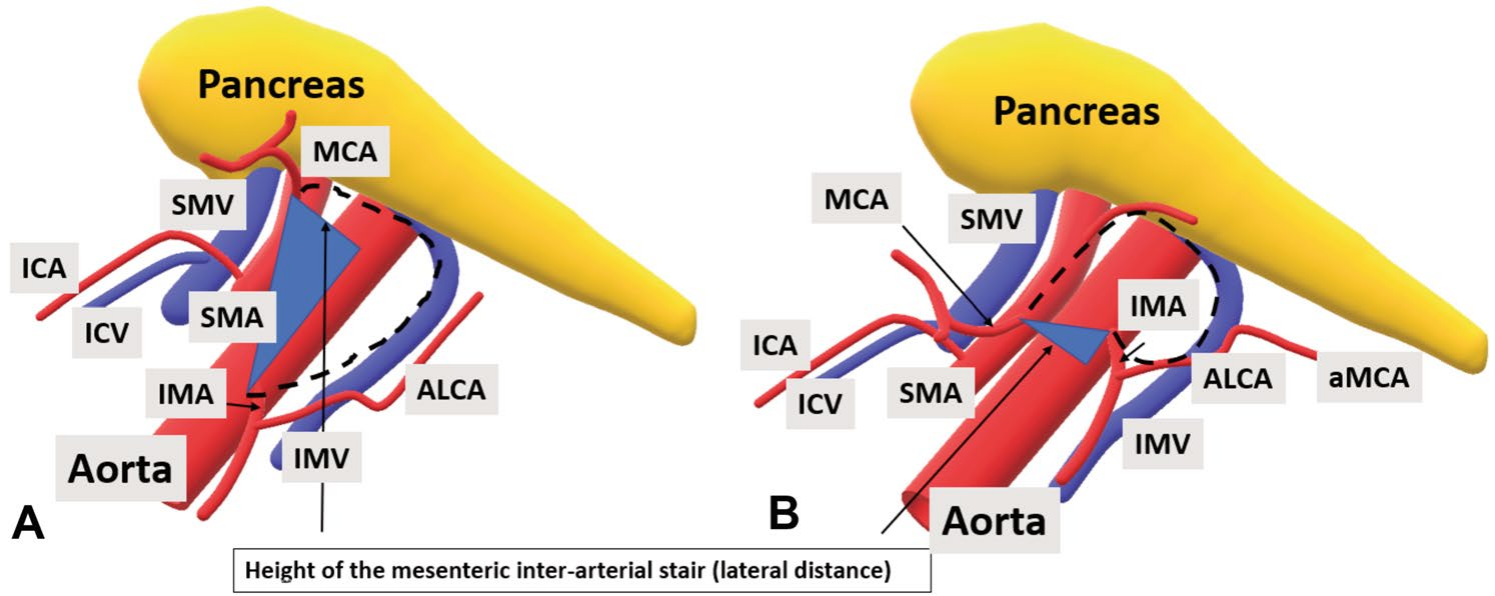
The mesenteric inter-arterial stair with positive (**A**) and negative (**B**) longitudinal distance. The dissection line is the black dashed line following IMV towards SMV, turning towards right at the lower border of pancreas (if IMV goes retropancreatically) and ending at the MCA origin. The stair is visualized as a triangle, with the height (lateral distance) indicated Vessels *MCA* middle colic artery, *aMCA* accessory middle colic artery, *ICA* ileocolic artery, *ICV* ileocolic vein,* SMV* superior mesenteric vein, *SMA* superior mesenteric artery, *IMA* inferior mesenteric artery *LCA* left colic artery

The third challenge is aMCA. The aMCA is present in about 1/3 of the patients (31.3% in our previously published data [9], which is similar to the 36.4% of patients reported by Bruzzi et.al [18]). It is more often present than the right colic artery proper which is present in 12.2% of patients [19], and aMCA runs directly towards the splenic flexure irrigating the area of the tumour. The trajectory of aMCA has been previously described as running equally along the body of the pancreas to the flexure or higher through the transverse mesocolon [9]. In this way it presents another possible pathway for direct lymphatic dissemination to the lymph nodes at its origin from SMA [17]. Despite low numbers, all patients with a cranial origin of IMA to that of MCA had an aMCA, while only one out of four patients had aMCA when the position was the inverse. Whether this can possibly be used as a sign to detect aMCA on the preoperative staging CT requires further research. In short, the surgeon should follow IMV or eventually the pancreatic notch when the IMV confluence is not accessible, to approach SMV and the MCA origin, in this way avoiding injury to the small bowel mesentery (follow the dashed line in Fig. 7). When the MCA origin lies caudal to the IMA origin, the dissection will be longer and the chance of identifying an aMCA higher. This emphasizes the importance of preoperative vascular mapping. Moreover, it seems possible that the arterial irrigation and venous drainage are highly variable and interchangeable depending on the stage of digestion [20–22] providing even more reason for lymphadenectomy along both arterial stalks.

Recent large series of comparatives studies of segmental resections of left flexure cancer versus extended resections (subtotal colectomy and extended left colectomy) have shown equal oncologic outcomes and rate of postoperative outcomes of these techniques, but segmental resections have been associated with shorted operative time [23–25]. Besides colon-sparing and avoiding dissection in close proximity to the pelvic nervous network would provide better functional outcomes. However, not all cases will be suitable to segmental resection [1, 25]. 3D reconstruction with awareness of anatomic findings of the present study will facilitate selection of patients to colon-sparing less invasive segmental resections and will enable to capture cases which still should be referred to extended resections due to specific features of the mesenteric vasculature.

Consequently, this study is a contribution towards a safer and more efficient strategy to perform an personalized oncological colon resection. 3D reconstruction of vascular anatomy with available on-screen virtual models, 2D printed images and/or a 3D physical model at surgery is a desirable prerequisite enabling the surgeon to provide safe and radical surgery, having anatomical knowledge about the mesenteric inter-arterial stair and the positioning of the major vessels before surgery. Nevertheless, operating surgeons that lack access to similar reconstructions can orient themselves on the anatomy through careful analysis of the preoperative CT scan in the coronal and sagittal planes [19, 26].

The limitation of this study is the relatively low number of patients, though many anatomical studies are performed with equal or a smaller number of patients. The interhuman anatomical variations are so diverse that there is a need for further studies with a larger study population [18, 27, 28]. Furthermore, all measurements have been done on ethnic North-Europeans and whether conclusions are universal, remains to be seen.

### Future developments

We predict that in the future 3D models will be available as a routine for all operations. The vascular maps will be presented on screen, paper, or as physical 3D models. In addition, there will be virtual models with possible use of VR goggles at surgery. All these aids will ultimately enable the surgeon to perform higher quality surgery through a personalized anatomical approach.

Segmental resections of left flexure cancer are going to be performed in higher rate due to their colon-sparing nature, thereby providing better functional outcomes while holding equal oncologic integrity as extended resections (subtotal colectomy and extended left colectomy). 3D reconstruction of mesenterial vasculature will enable personalized surgery.

## Conclusion

In conclusion we have presented three major challenges for the surgeon performing D3 extended mesenterectomy for splenic flexure cancer.

The first challenge is to address the difference in level between MCA and IMA without injuring the small bowel mesentery. This is the challenge of the mesenteric inter-arterial stair. To our knowledge this stair has not been described as such in the literature.

The second challenge is the different trajectories and confluences of IMV. Herein lies the solution to the first challenge. When the IMV trajectory is accessible, it enables safe access to the origin of the MCA and connects the two vascular areas. When the IMV trajectory is not accessible, the dissection should follow the caudal border of the pancreas.

The third challenge is the possible presence of aMCA representing another pathway for lymphatic dissemination.

We recommend preoperative CT 3D reconstructions to determine the nature of the patient’s mesenteric inter-arterial stair, the trajectory of the IMV and whether or not aMCA is present before D3 extended mesenterectomy for splenic flexure cancer is performed.
